# Circulation and Genetic Characterizations of Coronaviruses From Companion Animals in Chengdu, Southwest China: One-Year Postpandemic

**DOI:** 10.1155/tbed/7589098

**Published:** 2025-10-15

**Authors:** Linxuan Liao, Shanshan Wu, Yihang Xu, Mengxi Cao, Xiao Zhang, Liying Yi, Baochao Zhang, Jiayi Chen, Xin Xu, Xiaofang Pei

**Affiliations:** ^1^Department of Public Health Laboratory Sciences, West China School of Public Health and West China Fourth Hospital, Sichuan University, Chengdu 610041, Sichuan, China; ^2^Department of Quality Management and Planning, Ganzhou People's Hospital, Ganzhou 341000, Jiangxi, China

**Keywords:** codetection, companion animals, coronavirus, genetic characterizations, recombination

## Abstract

Coronaviruses (CoVs) can cross species barriers and endanger public health. Despite reports on their circulation and evolution in companion animals during the pandemic, postpandemic surveillance remains crucial. Therefore, during the first postpandemic year, 309 samples from 263 companion animals (cats and dogs) in Chengdu, China, were detected for CoVs using a universal assay based on Rdrp genes combined with one-generation sequencing. Four kinds of CoVs, including feline CoV (FCoV), canine CoV (CCoV), CRCoV, and SARS-CoV-2 (the first reported case of SARS-CoV-2 in a dog in mainland China, confirmed by viral nucleic acid detection and analysis), were detected with an overall positive rate of 21.7% (57/263); FCoV-I and CCoV-IIa were the dominant genotypes, and of these 57 positive cases, 71.9% (41/57) were in pets ≤12 months old. In CCoV-positive dogs, 72.2% (13/18) were coinfected with other viruses (primarily canine parvovirus [CPV], 76.9%; 10/13), while 13.9% (5/36) codetection with feline parvovirus (FPV). A 21-nt deletion in two *FCoV S* genes and a 145-nt deletion in one *FCoV ORF3abc* gene were identified, and recombination events at positions 919 and 1639 nt in two *S* genes were noticed. Notably, the amino acid variations in *FCoV* and *CCoV S* genes revealed distinct regional adaptations: FCoV strains showed unique substitutions (e.g., Ala/Ser129Leu) and a shift from RSRR to RARR furin cleavage motifs; CCoV strains in China exhibited significant differences from those in other countries. Phylogenetic analysis demonstrated that the *S* genes of FCoV and CCoV were closely related to those of the prevalent strains in China, whereas the *S* genes of CRCoV were closely related to that of human CoV (HCoV) OC43. These findings highlight the need for continued surveillance of CoV infection in companion animals (especially ≤12 months old) in the postpandemic era.

## 1. Introduction

Coronaviruses (CoVs) are enveloped, positive-stranded RNA viruses that infect mammals and birds, causing a range of diseases; the COVID-19 pandemic has heightened awareness of their prevalence and mutations in animals, including companion animals, due to the potential for cross-species transmission and the emergence of new variants [1].

The rising number of companion animals, coupled with close human–pet interactions, increases concerns on pathogen transmission from pets to owners. Cats and dogs can harbor various CoVs, potentially acting as reservoirs for zoonotic spillover. Notably, feline and canine CoVs (CCoV) have been detected in humans, such as the CCoV-HuPn-2018 strain in Malaysia and the HuCCoV_Z19Haiti strain in Haiti [2, 3]. And frequent mutations and recombination events in CoVs increase viral diversity and can lead to the emergence of new strains with altered virulence, tissue tropism, and host range [4]. For example, mutations in the *S*, *ORF3c*, and *ORF7b* genes of feline enteric coronavirus (FECV) enhance the virulence of feline infectious peritonitis virus (FIPV), shifting its tropism from enterocytes to monocyte/macrophage cells and causing severe systemic disease [5]. Similarly, SARS-CoV-2 continues to evolve, producing variants with higher transmissibility and altered pathogenicity [6]. Notably, porcine delta CoV (PDCoV) was identified in sick children in Haiti, with unique variations in the nsp15 and spike glycoprotein genes [7]. These findings highlight the need for ongoing surveillance and genetic characterization of emerging CoVs. Additionally, recombination events, which require coinfection and replication within the same host cell, can generate new strains with unique properties [8]. For instance, CCoV-IIb emerged from recombination between CCoV-II and porcine transmissible gastroenteritis virus, and feline CoV (FCoV)-II arose from recombination between FCoV-Ⅰ and CCoV-II [9–11]. Another example is the CCoV-A76 strain, which, due to S gene recombination, lost the ability to infect cat cells but gained tropism for dog and pig cell lines [12]. Therefore, continuous monitoring of mutations and recombination events in animal CoVs is crucial for public health, underscoring the need for integrated human–animal-environment surveillance systems. Li et al. [13] detected FCoV from 169 samples before the pandemic, and Santos et al. [14] screened for the CCoV, canine influenza virus, and SARS-CoV-2 from 86 dogs during the pandemic. However, these studies were conducted either before or during the pandemic; consequently, data from the postpandemic period remain limited.

Therefore, in this study, we conducted a molecular epidemiological investigation focusing on companion animals in southwest China using a pan-CoV semi-nested RT-PCR method. This approach detects and characterizes a broad spectrum of CoVs by amplifying conserved viral genome regions, aiding in identifying novel strains. Subsequently, we analyzed the genetic diversity and recombination of *S, N*, and accessory protein genes (*ORF3abc* and *ORF7ab*) by comparing them with pre-2023 strains. The aim of the study was to provide insights into the epidemiology, codetection, genetic evolution, and cross-species transmission of CoVs 1 year postpandemic in southwest China, which might contribute valuable data to ongoing CoV surveillance in companion animals and support the development of integrated human–animal-environment surveillance systems.

## 2. Materials and Methods

### 2.1. Sample Collection and Pretreatment

Between March 2023 and January 2024, anal and eye–nose–mouth swab samples from sick and healthy pets (cats and dogs) and ascites samples from cats that had developed peritonitis were collected at a veterinary hospital in Chengdu, an international metropolis located in the southwest part of China. In brief, samples were collected using sterile Dacron swabs handled with sterile gloves to prevent contamination and put in 3 mL viral transport media (Qingdao Hope Bio-Technology Co. Ltd., China) by trained veterinarians. The age, gender, breed, and clinical symptom information of the pets were also collected. Sample and data collections were approved by the pets' owners and veterinary hospital. After being transported to the laboratory on ice, centrifugation at 8000–10,000 rpm for 10 min at 4°C was conducted and 200 μL of the supernatant was used to extract the total nucleic acid with Virus DNA/RNA Extraction Kit II (Geneaid Biotech) according to the manufacturer's instructions. Thereafter, the extracted RNA was reverse transcribed into cDNA using the iScript cDNA Synthesis Kit (Bio-Rad, California, US).

### 2.2. Detection of CoVs

A previously established pan-CoV semi-nested RT-PCR assay, targeting the conserved Rdrp gene, was used to detect CoVs in samples [15] (Supporting Information 1: Table S1). For the initial reaction, a 25 µL reaction mixture was prepared, containing 3 μL cDNA, 12.5 μL 2 × Es Taq MasterMix (Dye) (Beijing Cowin Biotech Co., Ltd., China), 1 μL of each primer (10 μM), and 7.5 μL nuclease-free water. Subsequently, 2 μL of the product from the first reaction was used as the template for the semi-nested PCR reaction. The annealing temperatures for the first and second rounds of reactions were 55 and 53.6°C, respectively. The amplified products were sent for one-generation sequencing (Sangon Biotech, Shanghai, China), and the types of viruses were confirmed by using NCBI Blast comparison.

### 2.3. Detection of Other Enteric and Respiratory Viruses and Their Codetection With CoVs

To investigate codetection of CoVs with other viruses, seven other respiratory viruses and two enteric viruses were also detected in this study as previously described [16–21] (Supporting Information 1: Table S1). These included canine and feline influenza viruses, canine distemper virus (CDV), canine parainfluenza virus (CPIV), feline calicivirus (FCV), canine parvovirus (CPV), feline parvovirus (FPV), canine adenovirus type 2 (CAV-2), and feline herpesvirus type 1 (FHV-1).

### 2.4. CoVs Gene Amplification and Genotyping

Partial *S* genes were amplified for genotyping of FCoV and CCoV detected in this study as previously described [15, 22] (Supporting Information 1: Table S1). Additionally, the full length of *S*, *N*, *ORF3abc*, and *ORF7ab* genes from CoV-positive samples were amplified using primers referenced in the literature and self-designed primers. These primer sequences were listed in Supporting Information 1: Table S2 [15, 23–25]. The positive amplicons were subsequently sent to Sangon Biotech (Shanghai, China) for sequencing.

### 2.5. Sequence Analysis of *S*, *N*, *ORF3abc*, and *ORF7ab* Genes

The genetic sequences of these genes were assembled using the Seqman program in DNASTAR software (DNASTAR Inc., Madison, USA). The sequences of representative strains of domestic and international feline and CCoVs, human CoV (HCoV), and animal CoVs capable of infecting humans, which obtained before 2023, were retrieved from the NCBI nucleotide database as reference strains, and their information was listed in Supporting Information 1: Table S3. Multisequences alignments and homology analysis of nucleotides (nt) and deduced amino acids (aa) were performed using MEGA 11.0 software. For the calculation of mutation frequencies, FCoV C1Je (GenBank Accession Number DQ848678.1) and CCoV K378 (GenBank Accession Number KC175340.1) were used as reference strains, respectively. In the case of amino acid insertion or deletion, it was not included for the calculation of mutation frequencies.

### 2.6. Phylogenetic Analysis of *S* Gene

Neighbor-Joining (NJ) phylogenetic analysis of the *S* genes was performed based on the p-distance model and 1000 bootstrap replicates by comparison with reference sequences retrieved from the NCBI nucleotide database. The information for these reference sequences was listed in Supporting Information 1: Table S4. The phylogenetic trees were annotated using the Interactive Tree of Life (iTOL) software version 7 (https://itol.embl.de/).

### 2.7. Recombination Analysis

Recombination analysis on *S*, *N*, *ORF3abc*, and *ORF7ab* genes was performed using the recombination detection program RDP4 with RDP, 3Seq, GENECONV, Chimera, SiScan, MaxChi, and BootScan methods. The results were used for similarity plotting and bootscanning analyses with SimPlot software version 3.5.1.

### 2.8. Statistical Analysis

SPSS 25.0 software was used for statistical analysis. Samples were grouped according to gender, age, season, clinical status, and sample type, and the differences of CoV detection rates in different groups were compared by chi-square test, and a *p*-value of less than 0.05 was considered to be statistically significant. Further multiple comparison analysis was performed using partitions of the chi-square method, and the Bonferroni correction was applied to adjust the significance level (*α*).

## 3. Results

### 3.1. Detection Result Analysis for CoVs

A total of 309 samples from 263 companion animals (142 cats and 121 dogs) were collected in this study, including 224 anal swabs, 82 eye–nose–mouth swabs and 3 ascites samples, and the detection results were listed in Tables 1 and 2. The detailed information for each companion animal was also shown in Supporting Information 2: Table S5. Of the 263 animals, 57 were CoV-positive, with a rate of 21.7% (57/263). Among the 142 cats, the FCoV positive rate was 25.4% (36/142). And among the 121 dogs (one dog codetection of CCoV and CRCoV), the positive rate of CoVs in dogs was 17.4% (21/121), with those of CCoV, CRCoV, and SARS-CoV-2 being 14.0% (17/121), 3.3% (4/121), and 0.8% (1/121), respectively. It should be noticed that SARS-CoV-2 was detected in an eye–nose–mouth swab from a dog exhibiting pneumonia. To our knowledge, this is the first reported case of SARS-CoV-2 in a dog in this region, confirmed by nucleic acid detection and analysis. Besides, the detection rate of CoVs in anal swabs was 22.3% (50/224), which was significantly higher than that of eye–nose–mouth swabs (8.5%, 7/82; *p* < 0.01).

As shown in Table 1, a 71.9% (41/57) CoV-positive rate was observed in pets ≤12 months old, which was significantly higher than that in pets >12 months old (*p* < 0.01). The detection rates of CoVs in apparently healthy and sick cats and dogs were 14.3% (14/98) and 26.1% (43/165), respectively. The positive rate of CoVs in sick cats and dogs was higher than that in healthy ones (*p* < 0.05). Among the pets investigated, diarrhea, vomiting, and bloody stool were the main symptoms of CoV infection. Furthermore, statistical analysis on the detection rates of cats and dogs was performed separately (Supporting Information 1: Table S6). Significant differences were observed only between cats of different clinical statuses and among dogs of different age groups (*p* < 0.05). However, no statistically significant differences in CoV detection rates were observed across different genders and seasons.

Furthermore, from the FCoV and CCoV positive samples, 47 *S* genes were successfully obtained and sequenced. For FCoV, all 35 samples tested were positive for type I. For CCoV, nine samples were identified as type IIa, 1 sample as type I, and two samples were codetected with type I and type IIa; no type IIb was detected. These results indicated that FCoV-I and CCoV-IIa were the major infected genotypes of FCoV and CCoV in this region, respectively.

### 3.2. Detection Results for Other Enteric and Respiratory Viruses and Codetection With CoVs

As shown in Table 3, the detection rates of parvovirus in cats and dogs were 20.4% (29/142) and 28.1% (34/121), respectively. The detection rate of CPV from 163 dog samples was 28.2% (46/163), which notably exceeds that of CoVs (14.1%, 23/163). As shown in Table 4, among the 36 FCoV-positive samples, only concurrent with FPV was observed at a codetected rate of 13.9% (5/36). Among the 18 CCoV-positive samples, the overall codetection rate was 72.2% (13/18), and the highest codetection rate was with CPV at a rate of 55.6% (10/18). In addition, codetection of CCoV with CRCoV was observed in one dog sample. Moreover, CCoV-positive combining with other viruses in dogs (2 with CCoV + CPV + CAV-2, 1 with CCoV + CPV + CIV, and 1 with CCoV + CPV + CRCoV + CDV positive) were also observed, indicating the occurrence of multi-infection.

### 3.3. Amplification and Sequence Analysis of the CoV Strains

A total of 27 *S* genes, 28 *N* genes, 26 *ORF3abc* genes, and 31 *ORF7ab* genes were successfully amplified in their full-length forms. Fifteen *S* genes, 20 *N* genes, 16 *ORF3abc* genes, and 22 *ORF7ab* genes were obtained from FCoV-positive samples; while 10 *S* genes, 8 *N* genes, 10 *ORF3abc* genes, and 9 *ORF7ab* genes were obtained from CCoV-positive samples; and two *S* genes were obtained from CRCoV-positive samples. All sequences obtained from this study have been deposited in the GenBank database, and their accession numbers were listed in Supporting Information 1: Table S7, PV246283-PV246328 representing the gene sequences from FCoV or CCoV, whereas PV983458 representing the Rdrp gene sequence of SARS-CoV-2.

**Figure 1:**
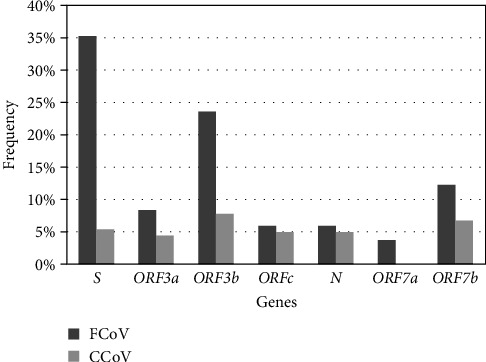
Frequencies of deduced amino acid mutations of the *S*, *N*, *ORF3abc*, and *ORF7ab* genes in FCoV and CCoV. FCoV C1Je (DQ848678.1) and CCoV K378 (KC175340.1) isolates were used as the reference strains for the definition of mutations, respectively. Dark gray indicates FCoV, light gray indicates CCoV.

As shown in Table 5, the nucleotide and deduced amino acid similarity percentages for the genes of *FCoV* and *CCoV* were presented. We found that the homology percentages of *S*, *N*, and *ORF7ab* genes for FCoV were much lower compared to those of CCoV except for *ORF3abc* genes. The similar pattern was also noticed for deduced amino acid homology of corresponding genes. Specifically, the *S* gene similarities of FCoV ranged from 81.33% to 88.98%, while for CCoV they ranged from 97.34% to 99.20%. The mutation frequencies of the deduced amino acid sequences of the *S*, *N*, *ORF3abc*, and *ORF7ab* genes in FCoV and CCoV were further calculated, respectively, and higher mutation rates were also noticed in FCoV (Figure 1). These results suggested that FCoV might have been more prone to mutation and evolution than CCoV.

**Figure 2:**
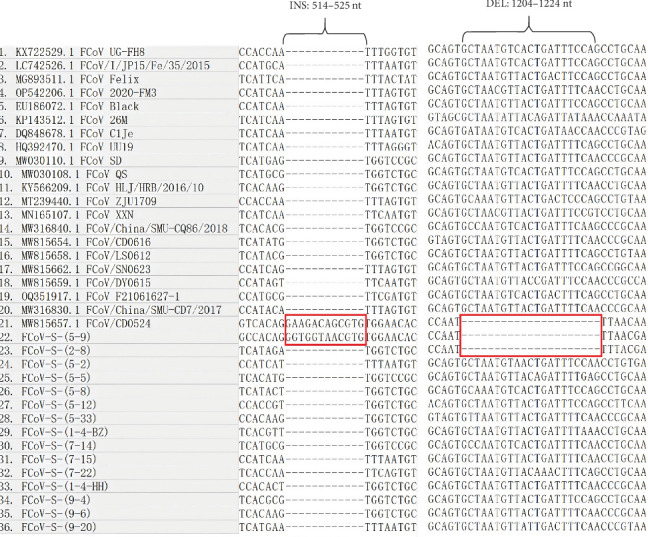
Sequence alignment of partial *S* genes between the identified FCoV strains and reference strains. The red rectangle showed the insertions and deletions of FCoV, compared to the reference strains.

Further nucleotide sequence alignment analysis, as shown in Figure 2, demonstrated a deletion between nt 1204–1224 in two FCoV S genes (FCoV-S-2-8, FCoV-S-5-9) resulting in a 7-aa deletion within the S1 subunit, and an insertion at positions 514–525 in FCoV-S-5-9 resulting in a 4-aa insertion (Supporting Information 1: Figure S1). On comparing the sequences of the *S* gene with those of the reference strains, only FCoV-S-5-9 shared the same deletion and insertion with the reference strain FCoV/CD0524, while FCOV-S-2-8 only had similar deletions without insertion. Notably, a 145 nt deletion spanning positions 139–283 of ORF3abc was observed in FCoV-ORF3abc-5-5, resulting in truncation of the ORF3a and a 42-aa contiguous deletion from the ORF3b start codon (Supporting Information 1: Figures S2 and S3). Additionally, a 3-aa deletion in the ORF7b protein of this strain was also noticed (Supporting Information 1: Figure S4). When comparing the *N* gene sequences of FCoV, no notable mutations were observed. By contrast, the detected CCoV sequences exhibited high conservation and the translated protein remained stable, indicating lower genetic variability than FCoV.

### 3.4. Comparison of Amino Acids Variations With Reference Strains

**Figure 3:**
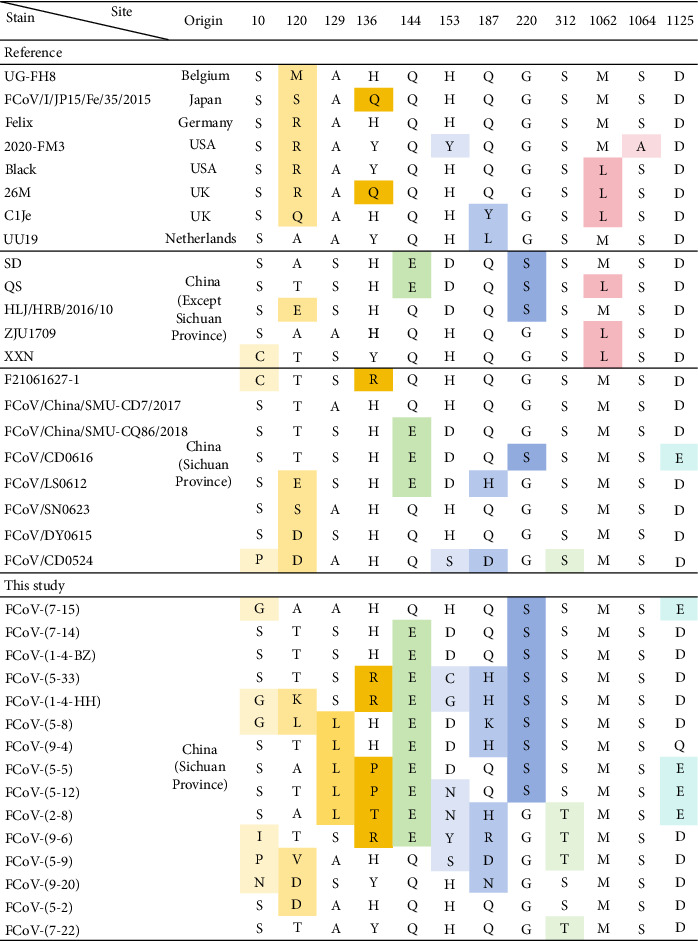
Comparison of amino acid variations in *FCoV S* genes. The same colors showed the mutations compared to reference strains.

**Figure 4:**
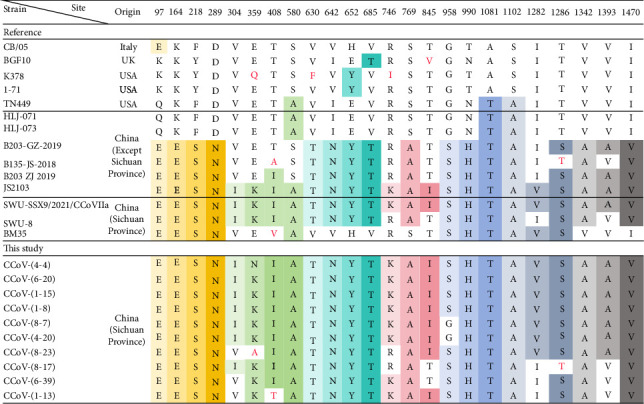
Comparison of amino acid variations in *CCoV S* genes. The same colors showed the mutations compared to reference strains. The letters shown in red indicated specific amino acid mutations in the proteins.

The amino acid variations of the *FCoV S* genes detected in this study were analyzed by comparing them with 21 selected reference strains of FCoV, which were collected before 2023. These reference strains consisted of eight from other countries and 13 from China, including eight strains that originated from Sichuan—the province where the present study was conducted. As shown in Figure 3, variations were highlighted in different colors, and a higher number of mutations were found in the *S* genes obtained in this study. Notably, the unique substitutions of Ala/Ser129Leu were found only in our sequences, whereas mutations like His/Tyr136Arg, Gln144Glu and Gly220Ser were observed only in the strains found in China. In particular, the mutations Ser312Thr and Asp1125Glu, which were similar to those in a reference strain from Sichuan Province, were observed more frequently in this study. Besides, the variations at position 120 showed the highest degree of diversity. Furthermore, the substitutions Met1062Leu and Ser1064Ala were not observed in the *FCoV S* gene sequences obtained in this study. For the furin cleavage site of the *FCoV S* gene, RSRR cleavage motifs were observed in most of the strains reported before the end of the COVID-19 pandemic, while more RARR cleavage motifs were observed in our sequences (Supporting Information 1: Figure S5). On the other hand, in the analysis of the *CCoV S* gene variation (as shown in Figure 4), the mutations in the virus found in China were quite similar to each other but significantly different from those reported in other countries.

### 3.5. Phylogenetic Analysis Based on *S* Gene

**Figure 5:**
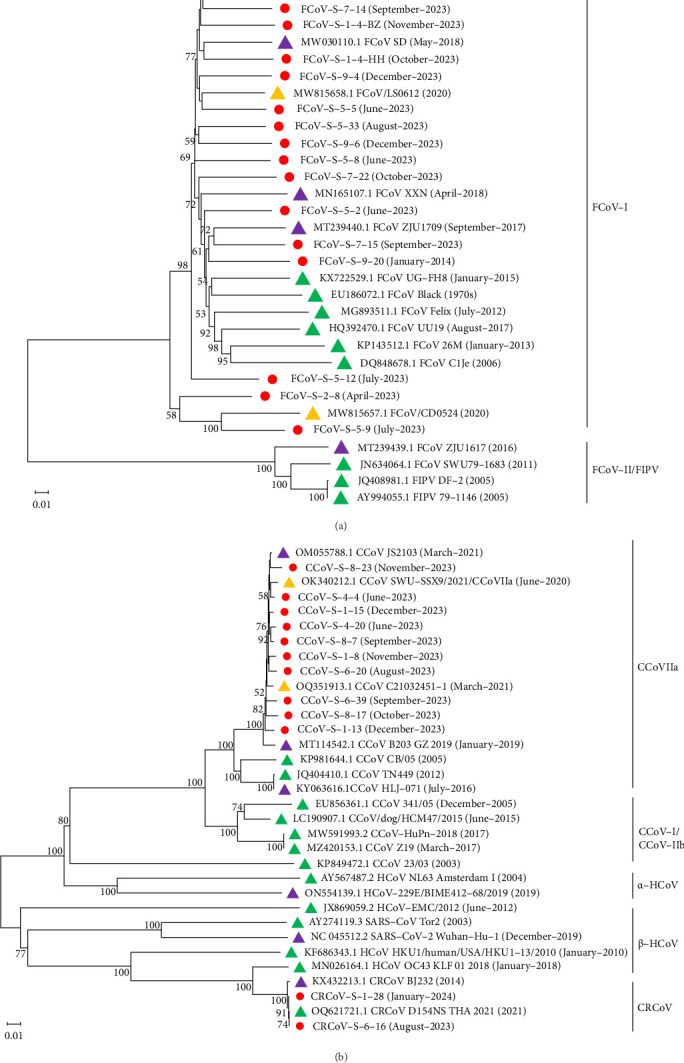
Phylogenetic analyses of FCoV and CCoV/CRCoV based on the complete *S* genes. (A) Phylogenetic tree of *FCoV S* genes; (B) Phylogenetic tree of *CCoV* and *CRCoV S* genes. Only bootstrap values ≥50% were shown. The red dots represented the sequences obtained in this study. The yellow, purple, and green triangles represented the reference strains from China (Sichuan Province), China (Except Sichuan Province), and other countries, respectively.

Phylogenetic analyses were performed on the *S* genes of FCoV, CCoV, and CRCoV obtained in this study together with reference sequences (obtained before 2023 with different geographical locations) from the NCBI database. As shown in Figure 5A, all the *FCoV S* genes obtained in this study clustered with those of FCoV-I reference strains, and most of them were closely related to those isolates from China. Among them, FCoV-S-5-9 clustered tightly with the Chinese reference strain FCoV/CD0524, and a close genetic relationship with that of FCoV-S-2-8 was also observed. In addition, the *S* genes of FCoV-S-7-15 and FCoV-S-1-4-HH were closely related to those of ZJU1709 and SD from China, respectively. Moreover, as shown in Figure 5B, the 10 *CCoV S* genes obtained in this study clustered with *CCoV-IIa S* genes and closely related to those of the prepandemic Chinese strains JS2103, SWU-SSX9, C21032451-1, and B203_GZ_2019. In addition, CRCoV-S-1-28 was more closely related to the Chinese strain BJ232 than to the Thai strain D154NS_THA_2021. Notably, the *S* genes of two CRCoV (CRCoV-S-1-28 and CRCoV-S-6-16) obtained in our study clustered with those of five β-HCoV strains and showed the closest relationship to the *S* gene of HCoV OC43.

### 3.6. Recombinant Analysis

**Figure 6:**
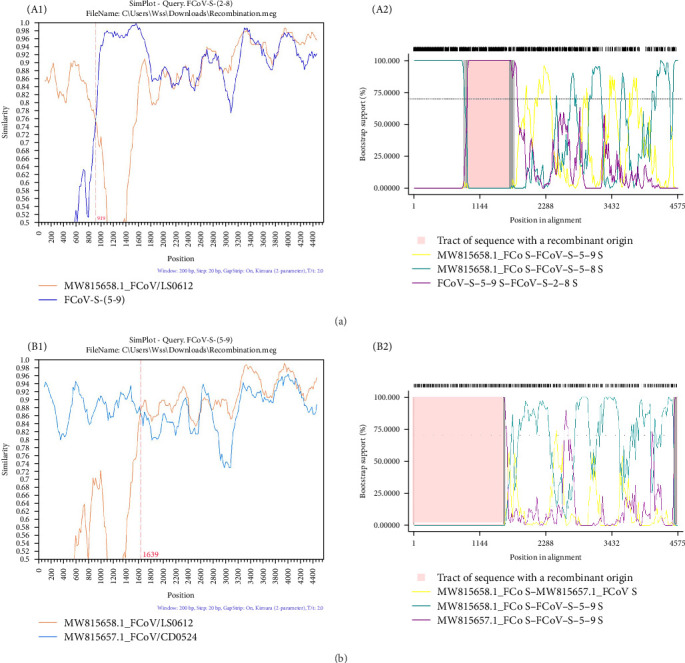
Analysis of the recombination sites for the *FCoV S* genes. (A) The SimPlot similarity (A1) and bootscanning analyses (A2) of FCoV-S-(2–8). In Figure 6A, the FCoV-S-(2–8) strain was designated as query strain. (B) The SimPlot similarity (B1) and bootscanning analyses (B2) of FCoV-S-(5–9). In Figure 6B, the FCoV-S-(5–9) strain was designated as query strain. Recombination breakpoints were indicated by red lines, with their positions labeled in red at the bottom. A similarity of 1.0 indicates 100% identity with the nucleotide sequence. A window size of 200 bp, a step size of 20 bp, and 100 replicates were used as the default settings.

After recombination analysis on the *S*, *N*, *ORF3abc*, and *ORF7ab* genes, recombination events in the *S* genes of two FCoV strains (FCoV-S-2-8 and FCoV-S-5-9) were observed. These were identified by all seven methods in RDP4, with *p*-values less than 10^−10^ and recombination scores higher than 0.6. As shown in Figure 6, the similarity plots and bootscanning analyses revealed that FCoV-S-2-8 was related to FCoV/LS0612 and FCoV-S-5-9, and that FCoV-S-5-9 was related to FCoV/LS0612 and FCoV/CD0524, which also confirmed the recombination events. Besides, the recombination positions were identified at 919 and 1639 nt, respectively.

## 4. Discussion

The number of companion animals has increased rapidly worldwide, reaching over 52.6 million dogs and 71.5 million cats in China alone, increases of 1.6% and 2.5% compared to 2023, respectively [26]. Companion animals in close contact with humans can be infected with various CoVs and may act as intermediate hosts for cross-species transmission. Therefore, continuous monitoring of CoVs and their mutations in these animals is crucial for identifying and mitigating potential health risks. Over a 1-year postpandemic period, we screened cat and dog samples for CoVs, characterized their genetic alterations, and compared them with prepandemic reference strains to track viral evolution.

In this study, a total of 309 samples collected from 263 companion animals were tested. Among 263 companion animals, 57 tested positive for CoV. Of these, 41 positives were in pets ≤12 months old, with a positive rate of 71.9%, suggesting that surveillance for CoVs in companion animals should focus on those ≤12 months old. Furthermore, among 263 cats and dogs, CoV detection rates were 26.1% (43/165) in symptomatic animals and 14.3% (14/98) in healthy ones. Similar results were also reported by Wu et al. [27]. Although 71.4% (10/14) of the CoV-positive healthy animals were ≤12 months old, subsequent analysis revealed no significant age-related difference in detection rates, likely because of the small number of positive samples. Additionally, 14.3% of healthy cats and dogs were CoV-positive, indicating asymptomatic infection or pathogen carriage; these animals may continuously shed virus, serving as mobile sources of infection and posing a potential public health risk [28]. In this study, statistically significant differences in the detection rates were found among different ages, clinical statuses, and sample types; however, nonrandom sampling, with a possible bias toward symptomatic animals, may have influenced these estimates. Nevertheless, the sample size in this study is relatively large; it may still provide references for further study. Besides, the overall positive rate of CoVs, as well as the positive rates for FCoV, CCoV, and CRCoV in this study, were lower than those reported in Beijing and central China [29, 30], but similar to those in some regions of Northeast China [31]. Furthermore, the major genotypes detected in this region were FCoV-I and CCoV-IIa, which are in line with those reported in most parts of China [32, 33], but different from those in Greece, Vietnam, and Japan [34–36].

During the COVID-19 pandemic, SARS-CoV-2 infections in companion animals have attracted attention. For example, Agüero et al. [37] and Sit et al. [38] reported SARS-CoV-2 infections in dogs through nucleic acid analysis in Chile and Hong Kong, China, respectively. In mainland China, several articles have reported SARS-CoV-2 antibodies positive in dogs, which implies the infection of this virus, but there has been no reported case through nucleic acid analysis [39, 40]. In this study, we reported the first case of SARS-CoV-2 in a dog in mainland China, confirmed by nucleic acid detection and analysis. We collected an eye–nose–mouth swab sample from a 1-year-old Shiba Inu dog presenting pneumonia symptoms (cough, lung rales, and dry retching) on September 1, 2023, and amplified the Rdrp gene. The amplified product was sequenced and confirmed as the SARS-CoV-2 Rdrp gene via NCBI BLAST. Subsequently, the sample was sent to the Chengdu Center for Disease Control and Prevention for next-generation sequencing. But the complete genomic sequence of SARS-CoV-2 was not obtained due to the poor sample quality. Besides, testing for other enteric and respiratory viruses was negative for this sample. The dog was treated with cephalosporin drugs, and recovered. During that period, the positive rate of the SARS-CoV-2 in influenza-like cases reported in mainland China by the Chinese Center for Disease Control and Prevention was 18.6%, suggesting that the virus might be transmitted from human to animal. However, as the samples of the pet' owner and environment were not collected, we did not obtain the direct evidence of human-to-animal transmission.

In this study, except CoVs, two enteric viruses and seven respiratory viruses were also detected, and codetection with CoVs was common, especially in dogs. Among FCoV-positive samples, only FCoV-FPV codetections were found. Whereas among 18 CCoV-positive samples, the positive rate of codetection was 72.2% (13/18), with CPV being the most frequent (55.6%, 10/18). Besides, a quadruple positive for CCoV, CPV, CRCoV, and CDV was detected in a dog. These findings were consistent with a 2022 study reporting that CCoV-CPV coinfection was the most common combination in China [41]. Although no human infections have been reported for CPV and FPV, both of them have broad host ranges and can infect human cells in vitro [42]. Therefore, further research on the zoonotic potential of these viruses is imperative.

In this study, the sequences of *S, N, ORF3abc*, and *ORF7ab* genes in FCoV and CCoV were further analyzed. The alignment results demonstrated that FCoV had lower similarity percentages (81.33%−88.98%) than CCoV (97.34%−99.20%). Besides, the mutation frequencies of the deduced amino acid sequences of S, N, ORF3abc, and ORF7ab in FCoV and CCoV were also compared by using FCoV C1Je (DQ848678.1) and CCoV K378 (KC175340.1) isolates as reference strains for defining mutations; these isolates were isolated in 2006 and 1978, respectively. We found that the mutation rates of these genes in FCoV were higher than those in CCoV. It is possible if the reference strain was isolated earlier, it might potentially contribute to a higher mutation rate; however, in this study, the reference strain for FCoV was isolated later than that for CCoV (2006 vs. 1978), yet higher mutation rates in FCoV were observed, which further suggested that FCoV was more susceptible to mutation and evolution, potentially leading to the emergence of novel subtypes and variants [5]. Furthermore, in this study, we identified a novel 145-nt deletion in ORF3abc gene of FCoV-ORF3abc-5-5, resulting in the truncation of ORF3a and ORF3b proteins. This deletion pattern has not been reported previously. This variant was detected in a 2-month-old male orange cat presenting with diarrhea, weight loss, anemia, and inflammation. Additionally, FCoV-S-2-8 and FCoV-S-5-9 were found to have a 7-aa deletion in the S1 subunit of their S proteins, which were detected in a 2-year-old cat with diarrhea and vomiting, and a 9-month-old cat with conjunctival redness and poor appetite, respectively. Moreover, FCoV-S-5-9 had a 5-aa insertion, sharing the same mutation pattern with the FCoV/CD0524 detected from Chengdu in 2020. It should be noticed that the impact of these mutations on FCoV virulence and tropism needs to be further studied.

Interestingly, after comparing *FCoV S* gene mutations with reference strains (eight from other countries and 13 from China, including eight from Sichuan) reported before 2023, more mutations and unique substitutions like Ala/Ser129Leu were observed in this study. Besides, mutations such as His/Tyr136Arg, Gln144Glu, and Gly220Ser were found exclusively in the Chinese strains selected. Notably, substitutions at positions 23,531 and 23,537 (aa 1062 and 1064; Figure 3) within the *S* gene have been associated with an increased likelihood of feline infectious peritonitis (FIP) development [43]. Unfortunately, in this study, we did not obtain the *S* gene fragments from FIP cases. In turn, these mutations were not observed in other *S* gene fragments obtained from non-FIP cases. Additionally, it has been reported that the S-A substitution could increase the effect on proteolysis by furin [44]. In this study, more RARR cleavage motifs were identified within the furin cleavage site of the *FCoV S* gene, underscoring the need for further investigation of this motif.

Phylogenetic analyses of the *S* gene demonstrated that most identified FCoV and CCoV strains clustered with Chinese reference strains reported before the ending of the COVID-19 pandemic, indicating stable transmission and adaptation in local animal populations. Notably, the *FCoV-S-5-9 S* gene was closely related to that of the FCoV/CD0524 strain from Chengdu in 2020 and shared a common phylogenetic origin with FCoV-S-2-8 and FCoV-S-5-12 S genes identified in this study. These findings suggest that these variant strains have been circulating in Chengdu since at least 2020. Furthermore, the S genes of the FCoV-S-7-15 and FCoV-S-1-4-HH were closely related to those of the Chinese reference strains ZJU1709 and SD, respectively, indicating distinct genetic lineages in the FCoV population. Besides, the two *CRCoV S* genes identified in this study were closely related to that of the HCoV OC43, suggesting a potential common ancestry [45]. This highlights the dynamic nature of CoV evolution and their ability to adapt to different hosts.

Recombination among CoVs drives new strain emergence, broadens host range and modulates virulence and tissue tropism, as demonstrated by CCoV-A76 and SARS-CoV-2 XD [12, 46]. Recombination within the *S* gene is frequently observed in both feline and CCoVs [47, 48]. Here, we identified two novel recombination events of *S* gene at positions 919 and 1639 nt (Figure 6), respectively, which were not reported previously. Therefore, continuous monitoring of recombinant events is imperative for understanding CoV genetic diversity and evolution.

However, this study has some limitations. First, we did not obtain samples from the environment, pet owners, or veterinary hospital staff to further support the presence of human-to-animal transmission. Future work should expand surveillance, including sampling, and continue monitoring. Second, this study only explored the *S, N, ORF3abc*, and *ORF7ab* genes. Since recombination can occur throughout the viral genome, particularly in ORF1ab, future studies utilizing complete genome sequences are necessary to gain a more comprehensive understanding of CoV evolution.

In conclusion, this is the first report on CoVs detection in companion animals during the first postpandemic year. We found that FCoV-I and CCoV-IIa are predominant in Chengdu, southwest of China, with frequent codetection with other viruses. Genetic variations, including deletions, mutations, and recombination events, are more observed in FCoV than in CCoV. The FCoV, CCoV, and CRCoV strains detected have a close relationship with those circulating in China before. Notably, a dog with SARS-CoV-2 positive confirmed with nucleic acid detection and analysis was reported for the first time in mainland China. Our findings highlight the importance of continuous investigation of CoVs in companion animals, especially in those ≤12 months old, to understand their genetic diversity and potential public health implications.

## Figures and Tables

**Table 1:** Correlation of the detection rate of CoVs with age, gender, season, and clinical status in animals.

Variables	Number of animals (*n* = 263)	Positive rate of CoV	Negative rate of CoV	*p*-Value
Gender				
Male	148	23.6% (35/148)	76.4% (113/148)	>0.05
Female	115	19.1% (22/115)	80.9% (93/115)	
Age				
≤12 months	145	28.3% (41/145)	71.7% (104/145)	<0.01
>12 months	118	13.6% (16/118)	86.4% (102/118)	
Season				
Spring	61	13.1% (8/61)	86.9% (53/61)	>0.05
Summer	85	18.8% (16/85)	81.2% (69/85)	
Autumn	57	33.3% (19/57)	66.7% (38/57)	
Winter	60	23.3% (14/60)	76.7% (46/60)	
Clinical status				
Healthy	98	14.3% (14/98)	85.7% (84/98)	<0.05
Presenting clinical symptoms	165	26.1% (43/165)	73.9% (122/165)	

**Table 2:** Detection rate of CoVs by sample type.

Sample type	Number of samples (*n* = 309)	Positive rate of CoV	Negative rate of CoV	*p*-Value
Anal swabs	224	22.3% (50/224)	77.7% (174/224)	<0.01
Eye–nose–mouth swabs	82	8.5% (7/82)	91.5% (75/82)	
Ascites samples^a^	3	33.3% (1/3)	66.7% (2/3)	

^a^Due to the small number of ascites samples, no statistical analysis of detection rates was conducted.

**Table 3:** Detection of other enteric and respiratory viruses.

Species	Pathogens	Positive rate of animals (*n* = 263)	Positive rate of anal swabs (*n* = 224)	Positive rate of eyes–nose–mouth swabs (*n* = 82)
Cat	FPV	20.4% (29/142)	21.5% (26/121)	13.6% (3/22)
	FHV-1	7.0% (10/142)	5.8% (7/121)	13.6% (3/22)
	FCV	0.7% (1/142)	0	4.5% (1/22)
	Feline influenza virus	0	0	0

Dog	CPV	28.1% (34/121)	27.2% (28/103)	30.0% (18/60)
	CAV-2	5.8% (7/121)	4.9% (5/103)	6.7% (4/60)
	CDV	4.1% (5/121)	2.9% (3/103)	6.7% (4/60)
	CPIV	5.0% (6/121)	1.9% (2/103)	6.7% (4/60)
	CIV	0.8% (1/121)	0	1.7% (1/60)

**Table 4:** Codetection with other viruses in CoV-positive samples.

CoV-positive samples	Type of codetection	Positive rate of codetection	Total positive rate of codetection
FCoV (*n* = 36)	FCoV + FPV	13.9% (5/36)	13.9% (5/36)

CCoV (*n* = 18)	CCoV + CPV	33.3% (6/18)	72.2% (13/18)
	CCoV + CPV + CAV-2	11.1% (2/18)	
	CCoV + CPV + CIV	5.6% (1/18)	
	CCoV + CPV + CRCoV + CDV	5.6% (1/18)	
	CCoV + CRCoV	5.6% (1/18)	
	CCoV + CDV	5.6% (1/18)	
	CCoV + CAV-2	5.6% (1/18)	

**Table 5:** Sequence similarity of *S*, *N*, *ORF3abc*, and *ORF7ab* genes in FCoV and CCoV.

Type of virus	Nucleotide and (amino acid) homology (%)			
	*S* genes	*N* genes	*ORF3abc* genes	*ORF7ab* genes
FCoV	81.33%−88.98% (83.00%−93.53%)	91.45%−95.40% (91.78%−97.35%)	93.77%−97.44%	90.76%−100.00%

CCoV	97.34%−99.20% (98.07%−99.66%)	98.87%−99.91% (99.48%−100.00%)	89.63%−99.91%	98.94%−99.84%

*Note:* Due to the fact that the *ORF3abc* and *ORF7ab* genes encode five accessory proteins, their amino acid similarity was not compared.

## Data Availability

All sequences obtained from this article have been deposited in the GenBank database under Accession Number: PV246283-PV246328 and PV983458. All data of this study are available from the corresponding author upon request.

## Supporting Information

Supporting Information 1 Table S1: Primers for the CoVs detection and genotyping, other intestinal and respiratory viruses detection. Table S2: Primers used for PCR amplification. Table S3: The reference strains found worldwide used for amino acid variation analysis. Table S4: The reference strains found worldwide used for phylogenetic analysis. Table S6: Correlation of the detection rate of CoVs with age, gender, season, and clinical status in cats and dogs. Table S7: NCBI Accession numbers for *FCoV, CCoV*, and *SARS-CoV-2* gene sequences obtained in this study. Figure S1: Partial deduced amino acid insertions and deletions of FCoV S proteins. Figure S2: Sequence alignment of partial *ORF3abc* genes between the identified FCoV strains and reference strains. Figure S3: Partial deduced amino acid deletions of FCoV ORF3a (A) and ORF3b (B) proteins. Figure S4: Partial deduced amino acid deletions of FCoV ORF7ab protein. Figure S5: The ‘R-S/A-RR' cleavage motif of FCoV S proteins.

Supporting Information 2 Table S5: The detailed information for cats and dogs included in this study.
