# Supplementary material for: Circulation and Genetic Characterizations of Coronaviruses From Companion Animals in Chengdu, Southwest China: One-Year Postpandemic
Source: Transbound Emerg Dis. 2025 Oct 15;2025:7589098. doi: 10.1155/tbed/7589098 (PMC12543499; doi:10.1155/tbed/7589098)
Supplement: Supporting Information 1 — Table S1: Primers for the CoVs detection and genotyping, other intestinal and respiratory viruses detection. Table S2: Primers used for PCR amplification. Table S3: The reference strains found worldwide used for amino acid variation analysis. Table S4: The reference strains found worldwide used for phylogenetic analysis. Table S6: Correlation of the detection rate of CoVs with age, gender, season, and clinical status in cats and dogs. Table S7: NCBI Accession numbers for FCoV, CCoV, and SARS-CoV-2 gene sequences obtained in this study. Figure S1: Partial deduced amino acid insertions and deletions of FCoV S proteins. Figure S2: Sequence alignment of partial ORF3abc genes between the identified FCoV strains and reference strains. Figure S3: Partial deduced amino acid deletions of FCoV ORF3a (A) and ORF3b (B) proteins. Figure S4: Partial deduced amino acid deletions of FCoV ORF7ab protein. Figure S5: The ‘R-S/A-RR' cleavage motif of FCoV S proteins. [file 7589098.f1.docx]

**Supplementary materials**

Table S1: Primers for the CoVs detection and genotyping, other intestinal and respiratory viruses detection

| Virus | Target gene | Primer name | Primer sequence (5'-3') | length (bp) | Annealing temperature (℃) | Reference |
| --- | --- | --- | --- | --- | --- | --- |
| CoVs | Rdrp (detection) | pan-CoV_outF | CCAARTTYTAYGGHGGITGG | Round 1:  670～673 | 55 | [15] |
|  |  | pan-CoV_R | TGTTGIGARCARAAYTCATGIGG |  |  |  |
|  |  | pan-CoV_inF | GGTTGGGAYTAYCCHAARTGTGA | Round 2:  599～602 | 53.6 |  |
|  |  | pan-CoV_R | TGTTGIGARCARAAYTCATGIGG |  |  |  |
| CCoV | S  (typing) | CECoV-Ⅰ-EL1F | CAAGTTGACCGTCTTATTACTGGTAG | 346 | 50 | [15] |
|  |  | CECoV-Ⅰ-EL1R | TCATATACGTACCATTATAGCTGAAGA |  |  |  |
|  |  | CECoV-Ⅱa-S5 | TGCATTTGTGTCTCAGACTT | 694 | 54 |  |
|  |  | CECoV-Ⅱa-S6 | CCAAGGCCATTTTACATAAG |  |  |  |
|  |  | CECoV-Ⅱb-CEPol-1 | TCTACAATTATGGCTCTATCAC | 370 | 54 |  |
|  |  | CECoV-Ⅱb-TGSP-2 | TAATCACCTAAMACCACATCTG |  |  |  |
| FCoV |  | FCoV-S1 | CCACACATACCAAGGCCA | Round 1:  702 | 50 | [22] |
|  |  | FCoV-S2 | CTTAATGCWTWTGTGTCTC |  |  |  |
|  |  | FCoV-Ⅰ-nIffles | CCTAGAAAGCCTCAGATGAGTG | Round 2:  360 | 48 | [15] |
|  |  | FCoV-Ⅰ-nIubs | CCAAGGCCATTTTACATA |  |  |  |
|  |  | FCoV-Ⅱ-nIcfs | CAGACCAAACTGGACTGTAC | Round 2:  211 |  |  |
|  |  | FCoV-Ⅱ-nIubs | CCAAGGCCATTTTACATA |  |  |  |
| Feline and canine influenza viruses | M_WHO_ | F | ATGAGYCTTYTAACCGAGGTCGAAACG | 244 | 48 | - |
|  |  | R | TGGACAAANCGTCTACGCTGCAG |  |  |  |
| FPV and CPV | VP2 | CP1-F | GTAAGCTTCCAGGAGACTTT | 671 | 56 | [16] |
|  |  | CP1-R | GTAAGCTTCGTCGTGTTCTT |  |  |  |
| CDV | H | CDV-F | CGAGTCTTTGAGATAGGGTT | 455 | 56 | [17] |
|  |  | CDV-R | CCTCCAAAGGGTTCCCATGA |  |  |  |
| CPIV | N | F | AGGAAATGTCTGACCACCA | 534 | 56 | [18] |
|  |  | R | GCCTACGGATTGTTCTCAG |  |  |  |
| CAV-2 | E3 | HA1 | CGCGCTGAACATTACTACCTTGTC | 1031 | 55 | [19] |
|  |  | HA2 | CCTAGAGCACTTCGTGTCCGCTT |  |  |  |
| FCV | ORF2 | Cali | AACCTGCGCTAACGTGCTTA | 926 | 57 | [20] |
|  |  | Cali2 | CAGTGACAATACACCCAGAAG |  |  |  |
|  |  | Cali3 | TGGTGATGATGAATGGGCATC | 477 | 57 |  |
|  |  | Cali4 | ACACCAGAGCCAGAGATAGA |  |  |  |
| FHV-1 | TK | FHV-F | GACGTGGTGAATTATCAGC | 288 | 56 | [21] |
|  |  | FHV-R | CAACTAGATTTCCACCAGGA |  |  |  |

Table S2: Primers used for PCR amplification

| Target virus | Primer name | Primer sequence (5'-3') | length (bp) | Annealing temperature (℃) | Reference |
| --- | --- | --- | --- | --- | --- |
| FCoV-S | FCoV-I-S1F | CTAAGGAAGGGTAAAATACTC | 1660 | 54 | [15] |
|  | FCoV-I-S1R | GCAGAATAAAAYCCATCTGGTA |  |  |  |
|  | FCoV-I-S2F | TATACAGATGTAATGGTGGATGT | 1616 | 55 |  |
|  | FCoV-I-S2R | CCATTRTAATATTGGGCACAAACTA |  |  |  |
|  | 1544-S3-F* | CACCYACAATAGGTAAGAGGTC | 1544 | 55 | - |
|  | 1544-S3-R* | CAAGTACAGCGTCAACAGAGA |  |  |  |
| CCoV-S | CECoV-II-S1F | GGTTGTTGGATTACTAAGGAA | 1599 | 55 | [15] |
|  | CECoV-II-S1R | ATTAGCAGTAAGTTGAGAACA |  |  |  |
|  | CECoV-II-S2F | TGGTTACAATTTCTTTAGCAC | 1713 | 56 |  |
|  | CECoV-II-S2F | ACCATGATGCCATTGTAATAT |  |  |  |
|  | S3F-1566* | GTTCTGCTATAGAAGACTTGCT | 1566 | 55 | - |
|  | S3R-1566* | CCATACAAGACCTGTAATGACT |  |  |  |
| CRCoV-S | CRCoV-S1F | TGGGTGTTGCGGTCATAATTAT | 1051 | 54 | [15] |
|  | CRCoV-S1R | TGCAATTGGCTGAACAGTGTAAC |  |  |  |
|  | CRCoV-S2F | ATGCTGTTGATTGTAAGAGTGA | 1088 | 54 |  |
|  | CRCoV-S2R | AATACCTTGGCCTGTAATAC |  |  |  |
|  | CRCoV-S3F | ATAGTGGTACTACTTGTTCTACTG | 1207 | 56 |  |
|  | CRCoV-S3R | TTGACTTAGCACATCCATGGT |  |  |  |
|  | CRCoV-S4F | GTTGCCTCCACTGCTCTCAGA | 1309 | 60 |  |
|  | CRCoV-S4R | TGCTCGACCTCAATGGGTCT |  |  |  |
|  | CCoV-EM-R* | TTCTTCCGACCACGAGAATTG |  |  | - |
| FCoV-N | FC-NF* | ACTCAACAGAAGCACGTACT | 1353 | 56 | - |
|  | FC-NR* | ACTACTGTCAGGYACACCTA |  |  |  |
| CCoV-N | NF | CTAAAGCTGGTGATTACTCAACAG | 1273 | 54 | [23] |
|  | NR | TAATAAATACAGCRTGGAGGAAAAC |  |  |  |
| FCoV-ORF3abc | S-TMD-1 | ATGGCCTTGGTA**Y**GTGTGGCT | 1426 | 55 | [24] |
|  | SM-r2 | ACCCAAATTGCAACA**Y**ACCATGC |  |  |  |
| CCoV-ORF3abc | CCoV-ORF3-1F* | GCCTTGGTATGTRTGGCTACT | 1517 | 54 | - |
|  | CCoV-ORF3R* | ACAACAYACCATGCATAGCTT |  |  |  |
|  | CCoV-ORF3-2F* | TTGTAGTACAGGTTGYTGTGG | 1452 | 54 |  |
|  | CCoV-ORF3R* | ACAACAYACCATGCATAGCTT |  |  |  |
| FCoV-ORF7ab | N2-sense | AACTTTGGTGATAGTGATCTCG | 1515 | 55 | [24] |
|  | p204 | GCTCTTCCATTGTTGGCTCGTC |  |  |  |
| CCoV-ORF7ab | 7ab-F | TGATCCTAAGAC**W**GGACAATTCC | 1313 | 54 | [25] |
|  | 7ab-R | AAATCTAGCATTGCCAAATCAAA |  |  |  |

* Self-designed primer pairs

The bases marked in red are slightly modified in this study.

Table S3: The reference strains found worldwide used for amino acid variation analysis

| Accession number | Strain | Genotype | Host | Country | Collection date |
| --- | --- | --- | --- | --- | --- |
| KX722529.1 | UG-FH8 | FCoV Ⅰ | Feline | Belgium | 2015 |
| MG893511.1 | Felix | FCoV Ⅰ | Feline | Germany | 2012 |
| KP143512.1 | 26M | FCoV Ⅰ | Feline | UK | 2013 |
| DQ848678.1 | C1Je | FCoV Ⅰ | Feline | UK | 2006 |
| HQ392470.1 | UU19 | FCoV Ⅰ | Feline | Netherlands | 2007 |
| EU186072.1 | Black | FCoV Ⅰ | Feline | USA | 1970s |
| LC742526.1 | FCoV/I/JP15/Fe/35/2015 | FCoV Ⅰ | Feline | Japan | 2015 |
| OP542206.1 | 2020-FM3 | FCoV Ⅰ | Feline | USA | 2020 |
| MW030110.1 | SD | FCoV Ⅰ | Feline | China | 2018 |
| MW030108.1 | QS | FCoV Ⅰ | Feline | China | 2018 |
| KY566209.1 | HLJ/HRB/2016/10 | FCoV Ⅰ | Feline | China | 2016 |
| MT239440.1 | ZJU1709 | FCoV Ⅰ | Feline | China | 2017 |
| MN165107.1 | XXN | FCoV Ⅰ | Feline | China | 2018 |
| MW316840.1 | FCoV/China/SMU-CQ86/2018 | FCoV Ⅰ | Feline | China | 2019 |
| MW815658.1 | FCoV/LS0612 | FCoV Ⅰ | Feline | China | 2020 |
| MW815657.1 | FCoV/CD0524 | FCoV Ⅰ | Feline | China | 2020 |
| MW815654.1 | FCoV/CD0616 | FCoV Ⅰ | Feline | China | 2020 |
| MW815662.1 | FCoV/SN0623 | FCoV Ⅰ | Feline | China | 2020 |
| MW815659.1 | FCoV/DY0615 | FCoV Ⅰ | Feline | China | 2020 |
| OQ351917.1 | F21061627-1 | FCoV Ⅰ | Feline | China | 2021 |
| MW316830.1 | FCoV/China/SMU-CD7/2017 | FCoV Ⅰ | Feline | China | 2017 |
| KP981644.1 | CB/05 | CCoV Ⅱa | Canine | Italy | 2005 |
| JQ404410.1 | TN449 | CCoV Ⅱa | Canine | USA | 2012 |
| KC175340.1 | K378 | CCoV Ⅱa | Canine | USA | 1978 |
| JQ404409.1 | 1-71 | CCoV Ⅱa | Canine | USA | 2012 |
| AY342160.1 | BGF10 | CCoV Ⅱa | Canine | UK | 2003 |
| KY063616.1 | HLJ-071 | CCoV Ⅱa | Canine | China | 2016 |
| MT114542.1 | B203_GZ_2019 | CCoV Ⅱa | Canine | China | 2019 |
| OM055788.1 | JS2103 | CCoV Ⅱa | Canine | China | 2021 |
| OK340212.1 | SWU-SSX9/2021/CCoVIIa | CCoV Ⅱa | Canine | China | 2020 |
| MT919267.1 | BM35 | CCoV Ⅱb | Canine | China | 2019 |
| KY063618.2 | HLJ-073 | CCoV Ⅱa | Canine | China | 2016 |
| MT114544.1 | B135-JS-2018 | CCoV Ⅱa | Canine | China | 2018 |
| MT114541.1 | B203 ZJ 2019 | CCoV Ⅱa | Canine | China | 2019 |
| ON107244.1 | SWU-8 | CCoV Ⅱa | Canine | China | 2020 |

Table S4: The reference strains found worldwide used for phylogenetic analysis

| Accession number | Strain | Genotype | Host | Country | Collection date |
| --- | --- | --- | --- | --- | --- |
| KX722529.1 | UG-FH8 | FCoV Ⅰ | Feline | Belgium | 2015 |
| MG893511.1 | Felix | FCoV Ⅰ | Feline | Germany | 2012 |
| KP143512.1 | 26M | FCoV Ⅰ | Feline | UK | 2013 |
| DQ848678.1 | C1Je | FCoV Ⅰ | Feline | UK | 2006 |
| HQ392470.1 | UU19 | FCoV Ⅰ | Feline | Netherlands | 2007 |
| EU186072.1 | Black | FCoV Ⅰ | Feline | USA | 1970s |
| MW030110.1 | SD | FCoV Ⅰ | Feline | China | 2018 |
| MW030108.1 | QS | FCoV Ⅰ | Feline | China | 2018 |
| KY566209.1 | HLJ/HRB/2016/10 | FCoV Ⅰ | Feline | China | 2016 |
| MT239440.1 | ZJU1709 | FCoV Ⅰ | Feline | China | 2017 |
| MN165107.1 | XXN | FCoV Ⅰ | Feline | China | 2018 |
| MW316840.1 | FCoV/China/SMU-CQ86/2018 | FCoV Ⅰ | Feline | China | 2019 |
| MW815658.1 | FCoV/LS0612 | FCoV Ⅰ | Feline | China | 2020 |
| MW815657.1 | FCoV/CD0524 | FCoV Ⅰ | Feline | China | 2020 |
| JN634064.1 | FCoV WSU 79-1683 | FCoV Ⅱ | Feline | USA | 2011 |
| MT239439.1 | FCoV ZJU1617 | FCoV Ⅱ | Feline | China | 2016 |
| JQ408981.1 | FIPV DF-2 | FIPV | Feline | USA | 2005 |
| AY994055.1 | FIPV 79-1146 | FIPV | Feline | USA | 2005 |
| KP849472.1 | 23/03 | CCoV Ⅰ | Canine | Italy | 2003 |
| KP981644.1 | CB/05 | CCoV Ⅱa | Canine | Italy | 2005 |
| JQ404410.1 | TN449 | CCoV Ⅱa | Canine | USA | 2012 |
| KY063616.1 | HLJ-071 | CCoV Ⅱa | Canine | China | 2016 |
| MT114542.1 | B203_GZ_2019 | CCoV Ⅱa | Canine | China | 2019 |
| OM055788.1 | JS2103 | CCoV Ⅱa | Canine | China | 2021 |
| OK340212.1 | SWU-SSX9/2021/CCoVIIa | CCoV Ⅱa | Canine | China | 2020 |
| OQ351913.1 | C21032451-1 | CCoV Ⅱa | Canine | China | 2021 |
| EU856361.1 | 341/05 | CCoV Ⅱb | Canine | Italy | 2005 |
| LC190907.1 | CCoV/dog/HCM47/2015 | CCoV Ⅱb | Canine | Viet Nam | 2015 |
| MW591993.2 | CCoV-HuPn-2018 | CCoV Ⅱb | Human | Malaysia | 2017 |
| MZ420153.1 | Z19 | CCoV Ⅱb | Human | Haiti | 2017 |
| OQ621721.1 | D154NS THA 2021 | CRCoV | Canine | Thailand | 2021 |
| KX432213.1 | BJ232 | CRCoV | Canine | China | 2014 |
| AY567487.2 | NL63 Amsterdam I | α-HCoV | Human | Netherlands | 2004 |
| ON554139.1 | HCoV-229E/BIME412-68/2019 | α-HCoV | Human | China | 2019 |
| MN026164.1 | O43 KLF 01 2018 | β-HCoV | Human | Kenya | 2018 |
| KF686343.1 | HKU1/human/USA/HKU1-13/2010 | β-HCoV | Human | USA | 2010 |
| JX869059.2 | HCoV-EMC/2012 | β-HCoV | Human | Netherlands | 2012 |
| AY274119.3 | SARS-CoV Tor2 | β-HCoV | Human | Canada | 2003 |
| NC_045512.2 | SARS-CoV-2 Wuhan-Hu-1 | β-HCoV | Human | China | 2019 |

Table S6: Correlation of the detection rate of CoVs with age, gender, season, and clinical status in cats and dogs

| Variables | Cats | | | *p* value | Dogs | | | *p* value |
| --- | --- | --- | --- | --- | --- | --- | --- | --- |
|  | Numbers (n=142) | Positive rate of CoV | Negative rate of CoV |  | Numbers (n=121) | Positive rate of CoV | Negative rate of CoV |  |
| Gender | | | | | | | | |
| Male | 77 | 24.7% (19/77) | 75.3% (58/77) | ＞0.05 | 71 | 22.5% (16/71) | 77.5% (55/71) | ＞0.05 |
| Female | 65 | 26.2% (17/65) | 73.8% (48/65) |  | 50 | 10.0% (5/50) | 90.0% (45/50) |  |
| Age | | | | | | | | |
| ≤12 months | 84 | 26.2% (22/84) | 73.8% (62/84) | ＞0.05 | 61 | 31.1% (19/61) | 68.9% (42/61) | ＜0.01 |
| >12 months | 58 | 22.4% (13/58) | 77.6% (45/58) |  | 60 | 5.0% (3/60) | 95.0% (57/60) |  |
| Season | | | | | | | | |
| Spring | 32 | 18.8% (6/32) | 81.2% (26/32) | ＞0.05 | 29 | 6.9% (2/29) | 93.1% (27/29) | ＞0.05 |
| Summer | 42 | 23.8% (10/42) | 76.2% (32/42) |  | 43 | 14.0% (6/43) | 86.0% (37/43) |  |
| Autumn | 30 | 33.3% (10/30) | 66.7% (20/30) |  | 27 | 33.3% (9/27) | 66.7% (18/27) |  |
| Winter | 38 | 26.3% (10/38) | 73.7% (28/38) |  | 22 | 18.2% (4/22) | 81.8% (18/22) |  |
| Clinical status | | | | | | | | |
| Healthy | 59 | 15.3% (9/59) | 84.7% (50/59) | ＜0.05 | 39 | 12.8% (5/39) | 87.2% (34/39) | ＞0.05 |
| Presenting clinical symptoms | 83 | 32.5% (27/83) | 67.5% (56/83) |  | 82 | 19.5% (16/82) | 80.5% (66/82) |  |

Table S7: NCBI Accession numbers for FCoV, CCoV and SARS-CoV-2 gene sequences obtained in this study

| Accession number | Strain | Gene (s) | Accession number | Strain | Gene (s) |
| --- | --- | --- | --- | --- | --- |
| PV246287 | FCoV-1-4-HH | S, ORF3abc, N, ORF7ab | PV246309 | FCoV-5-33 | S, ORF3abc |
| PV246288 | FCoV-2-8 | S, ORF3abc, N, ORF7ab | PV246310 | FCoV-9-6 | S, ORF3abc |
| PV246289 | FCoV-5-2 | S, ORF3abc, N, ORF7ab | PV246311 | CCoV-4-20 | S, ORF3abc |
| PV246290 | FCoV-5-5 | S, ORF3abc, N, ORF7ab | PV246312 | CCoV-8-17 | S, ORF3abc |
| PV246291 | FCoV-5-8 | S, ORF3abc, N, ORF7ab | PV246313 | FCoV-5-31 | N, ORF7ab |
| PV246292 | FCoV-5-9 | S, ORF3abc, N, ORF7ab | PV246314 | FCoV-7-30 | N, ORF7ab |
| PV246293 | FCoV-5-12 | S, ORF3abc, N, ORF7ab | PV246315 | FCoV-9-4 | N, ORF7ab |
| PV246294 | FCoV-7-14 | S, ORF3abc, N, ORF7ab | PV246316 | FCoV-9-13 | N, ORF7ab |
| PV246295 | FCoV-7-15 | S, ORF3abc, N, ORF7ab | PV246325 | FCoV-5-33 | N, ORF7ab |
| PV246296 | FCoV-7-22 | S, ORF3abc, N, ORF7ab | PV246326 | FCoV-7-26 | N, ORF7ab |
| PV246297 | FCoV-9-20 | S, ORF3abc, N, ORF7ab | PV246327 | FCoV-9-6 | N, ORF7ab |
| PV246298 | CCoV-4-4 | S, ORF3abc, N, ORF7ab | PV246328 | CCoV-4-20 | N, ORF7ab |
| PV246299 | CCoV-6-39 | S, ORF3abc, N, ORF7ab | PV246305 | FCoV-5-23 | ORF7ab |
| PV246300 | CCoV-8-7 | S, ORF3abc, N, ORF7ab | PV246306 | FCoV-7-20 | ORF7ab |
| PV246301 | CCoV-8-23 | S, ORF3abc, N, ORF7ab | PV246307 | FCoV-7-23 | ORF7ab |
| PV246302 | CCoV-1-8 | S, ORF3abc, N, ORF7ab | PV246308 | CCoV-6-20 | ORF7ab |
| PV246303 | CCoV-1-13 | S, ORF3abc, N, ORF7ab | PV246283 | FCoV-9-4 | S |
| PV246304 | CCoV-1-15 | S, ORF3abc, N, ORF7ab | PV246284 | CCoV-6-20 | S |
| PV246324 | FCoV-5-20 | ORF3abc, N, ORF7ab | PV246285 | CRCoV-1-28 | S |
| PV246323 | FCoV-1-4-BZ | S, ORF3abc, N | PV246286 | CRCoV-6-16 | S |
| PV246321 | FCoV-5-23 | ORF3abc | PV983458 | SARS-CoV-2/dog/CHN/6-31/2023 | Rdrp |
| PV246322 | CCoV-8-13 | ORF3abc |  |  |  |


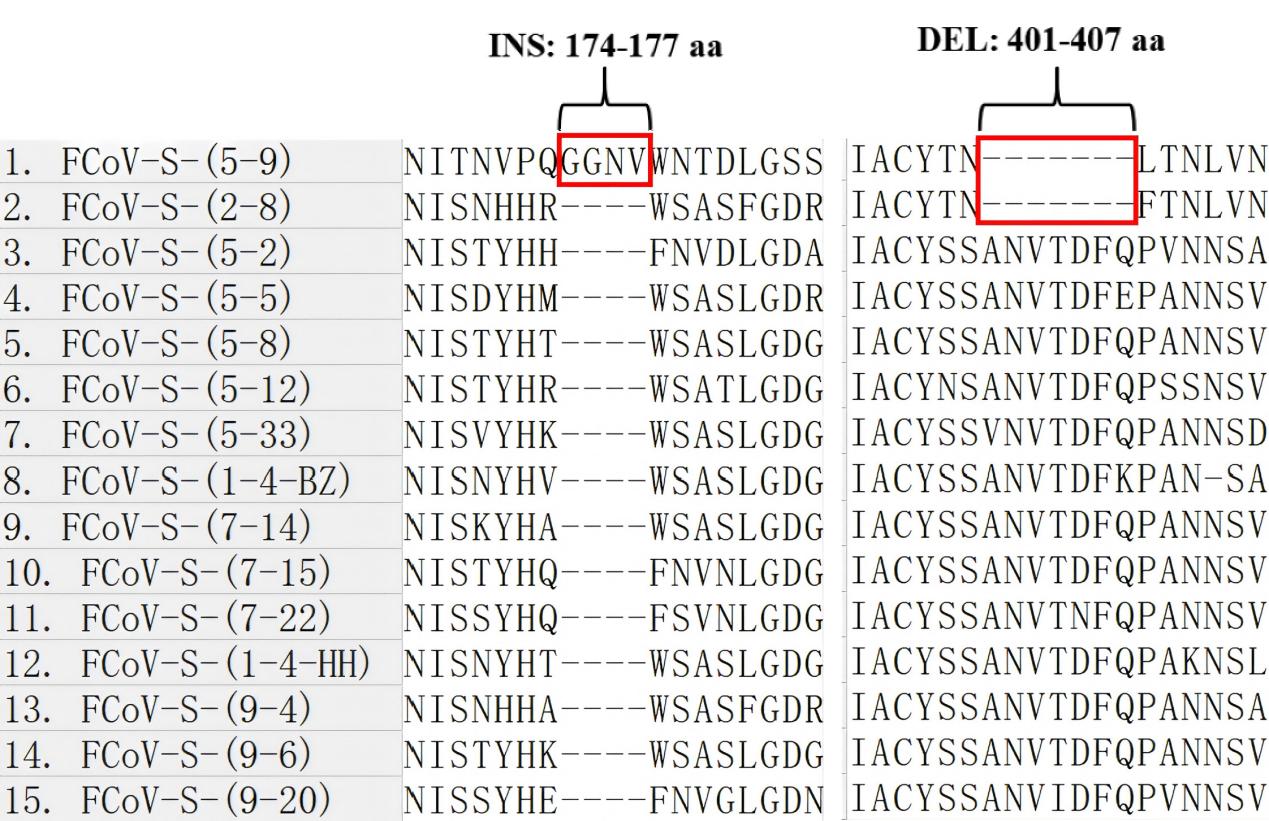


Figure S1: Partial deduced amino acid insertions and deletions of FCoV S proteins. The red rectangle showed four deduced amino acid insertions of the FCoV-S-5-9 S protein, and seven deduced amino acid deletions of the FCoV-S-2-8 and FCoV-S-5-9 S proteins.


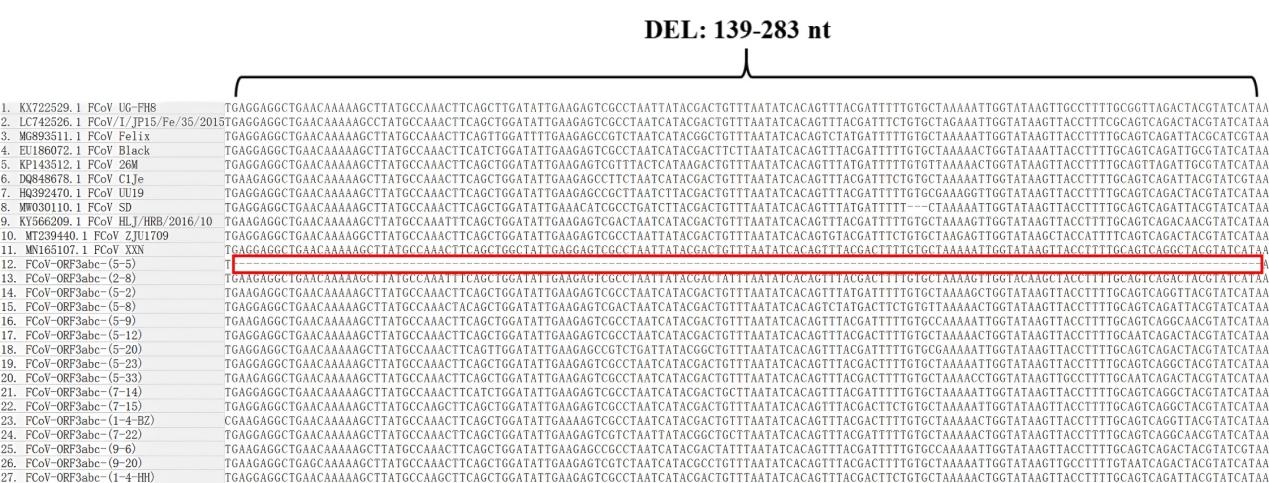
Figure S2: Sequence alignment of partial ORF3abc genes between the identified FCoV strains and reference strains. The red rectangle showed 145 consecutive deletions of the FCoV-S-(5-5) ORF3abc gene at positions 139-283.


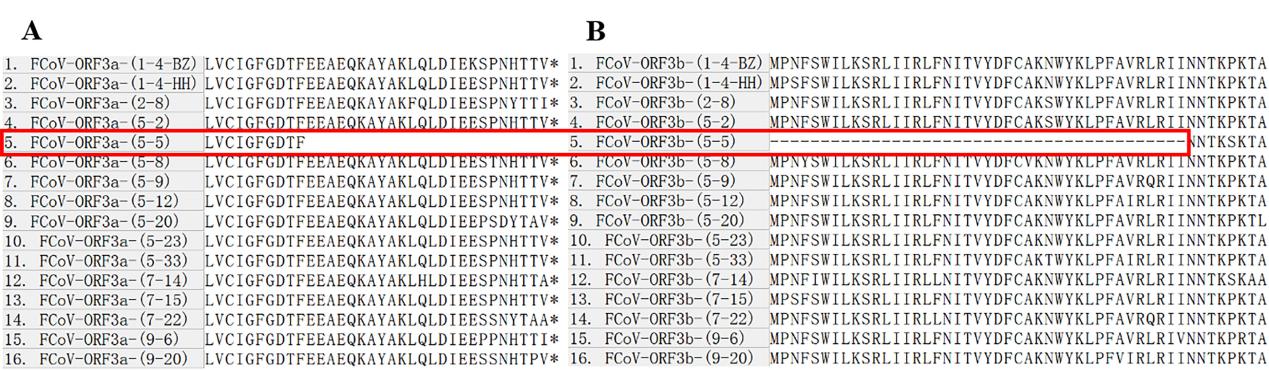


Figure S3: Partial deduced amino acid deletions of FCoV ORF3a (A) and ORF3b (B) proteins. The red rectangle showed deduced amino acid deletions of the FCoV-S-5-5 ORF3abc protein.


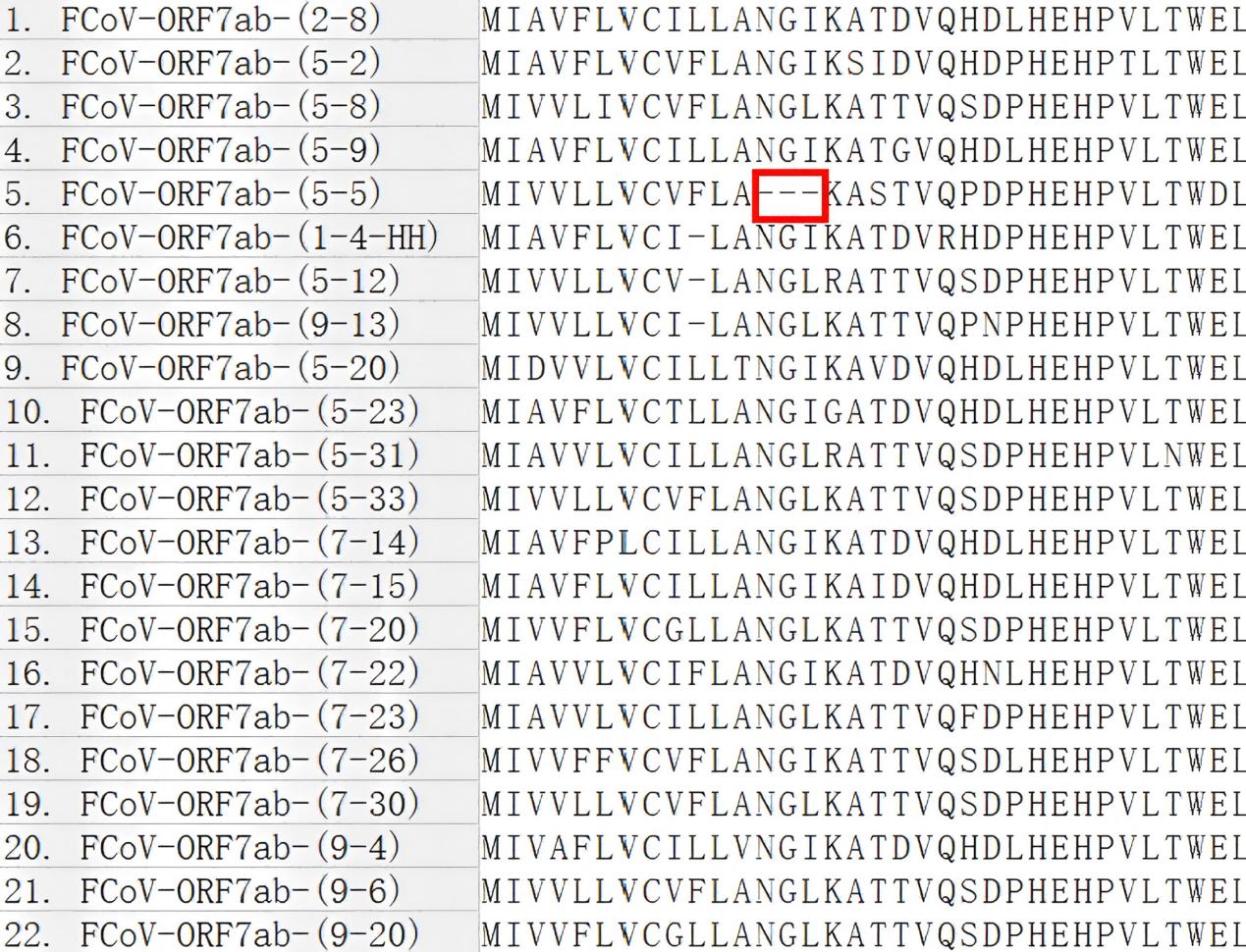


Figure S4: Partial deduced amino acid deletions of FCoV ORF7ab protein. The red rectangle showed deduced amino acid deletions of the FCoV-ORF7ab-5-5 ORF7abc proteins.


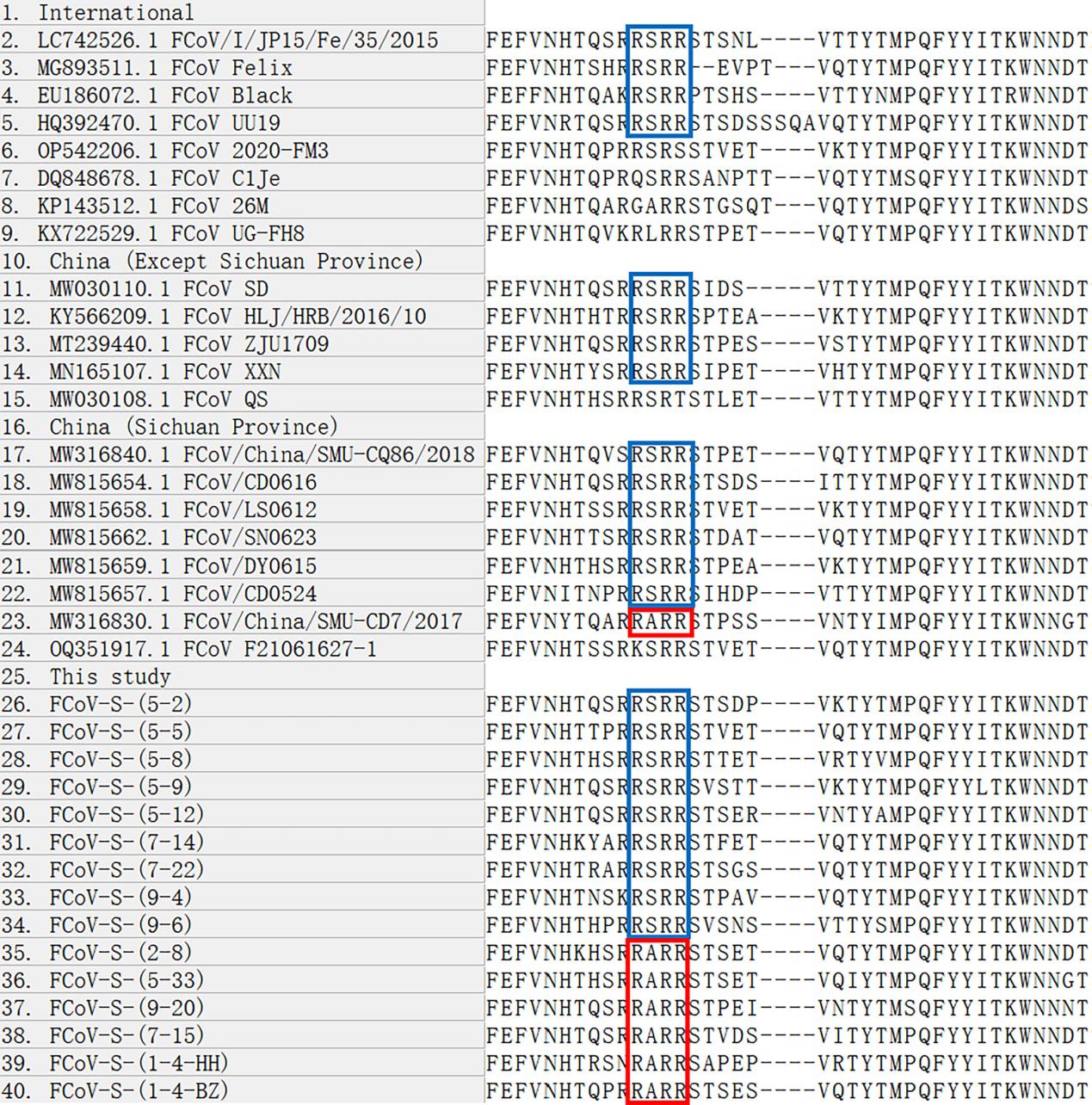


Figure S5: The ‘R-S/A-RR’ cleavage motif of FCoV S proteins. The red rectangle showed the RSRR cleavage motifs of the reference strain and the strain obtained in this study, and the blue rectangle showed the RARR cleavage motifs of the reference strain and the strain obtained in this study.
